# Disordered eating in early childhood: DRD4 and DAT1 gene polymorphisms and quality of mother–child interaction

**DOI:** 10.1007/s40519-022-01408-4

**Published:** 2022-05-05

**Authors:** Esterina Pascale, Silvia Cimino, Luca Cerniglia, Arturo Bevilacqua

**Affiliations:** 1 Department of Medico-Surgical Sciences and Biotechnologies, Sapienza University of Rome, Rome, Italy; 2 Department of Dynamic, Clinical Psychology and Health Studies, Sapienza University of Rome, Rome, Italy; 3 Faculty of Psychology, International Telematic University Uninettuno, 00186 Rome, Italy; 4 Department of Dynamic, Clinical Psychology and Health Studies, Systems Biology Group Lab, Sapienza University of Rome, The Experts Group on Inositols in Basic and Clinical Research (EGOI), Via dei Marsi 78, 00185 Rome, Italy

**Keywords:** Eating disturbances, Children, Parent–child interaction, DRD4 rs1805186, DAT1 rs28363170

## Abstract

**Purpose:**

Eating disturbances are complex heritable conditions that can be influenced by both genetic and environmental factors but are poorly studied in early development. The aim of this research was to investigate the association of genetic polymorphisms within dopaminergic pathways with early feeding problems.

**Methods:**

We analyzed the presence of VNTR polymorphisms of DRD4 (rs1805186) and DAT1 (rs28363170) in overeating (*N* = 45), undereating (*N* = 48) and control (*N* = 44) young children. We also assessed presence of externalizing, internalizing and dysregulation symptoms by the Child Behavior Checklist and quality of mother–child interactions during feeding by the Italian adaptation of the Scale for the Assessment of Feeding Interaction, respectively.

**Results:**

Both polymorphisms were associated with children’s eating behavior, psychological symptoms and quality of interaction with their mothers, suggesting that: (a) the DRD4 4-repeat allele behaves as a protective factor, the 2-repeats and 7-repeats alleles as risk factors, for undereating behavior, the general quality of mother–child interaction and internalizing, externalizing and dysregulated symptoms; and (b) the DAT1 9-repeats allele behaves as a protective factor, the 10-repeats allele as a risk factor, for overeating behavior, the general quality of mother–child interaction, internalizing, externalizing and dysregulated symptoms. Finally, a gene x gene interaction is suggested between the DAT1 9-repeat or 10-repeat allele and the DRD4 4-repeat allele.

**Conclusions:**

Our results suggest a role for DRD4 and DAT1 in an early susceptibility to eating disturbances.

**Level of evidence III:**

Evidence obtained from well-designed case–control analytic study.

## Introduction

Eating disturbances (EDs) in early childhood affect approximately 25% of children with typical development and 80% of children with developmental disorders [1, 2]. They may resolve spontaneously during development or produce more structured and severe problems for which late interventions would harm their outcome [3]. These difficulties are studied by focusing on both the child who, independently from food availability, feeds inadequately [undereating (UE)] or excessively [overeating (OE)] [4], and possible early-arising maladaptive aspects of the parent–child relationship with associated low-quality interactions during feeding and play [5, 6]. During feeding, a negative maternal affective state and interactions characterized by conflictual, non-collaborative, and non-empathetic communication, may contribute to children’s food refusal and to a negative emotional climate. Maternal intrusiveness or withdrawal in interactive exchanges with the child may depend on a lack of pleasure in reciprocal interactions [7], probably produced by an impairment (s) in the neurobiological circuits of reward [8]. Conversely, parents’ attention and sensitivity to the needs of the child, including the recognition of cues of hunger and satiety from breastfeeding and weaning, appear as key factors in the prevention of early EDs [9–11]. Observational procedures for the assessment of the quality of mother–child interactions during feeding such as the Scale for the Assessment of Feeding Interaction [12] have been used to evaluate at-risk patterns of caregiver–children’s interactions associated with children’s low ability to regulate affects and behavior, frequently correlated with EDs.

A genetic influence on EDs has been established as well [13, 14]. Maladaptive relational indicators may have a genetic basis, may emerge early during the first year of life and constitute a very significant risk factor for the appearance of children’s disordered eating [15]. Genetic studies, supported by the identification of neurotransmitters, hormones and peptides possibly regulating eating behavior and involved in the pathophysiology of EDs, have been investigated, among others, genes encoding factors responsible for dopamine (DA) dynamics, including receptors, membrane transporters and metabolizing enzymes [16–18], due to DA activity in subcortical and hypothalamic circuits controlling brain reward systems, appetite and satiety pathways [19]. Altered functioning of DA circuitry is involved in psychopathology, including abnormal feeding [20], and may be associated with the dysregulation of adaptive emotions and to internalized states of hypervigilance, withdrawal and inhibition, depression, anxiety and attachment insecurity [21].

Despite the large amount of information gathered on the genetics of major EDs in adults, data on young children are scarce or completely lacking. Indeed, studies on genetic variables associated with children’s EDs would be particularly interesting in Developmental Psychology, allowing the proposal of paradigm (s) intertwining psychological and biological variables.

In an initial attempt to close this gap, we have investigated the possible genetic and psychopathological correlates of EDs in young Italian children with diagnoses of overeating or undereating or with normal feeding behavior.

Genetic analysis was focused on variable number of tandem repeats (VNTR) polymorphisms of genes encoding for the dopamine D4 receptor (DRD4) and dopamine transporter (DAT1). Among DA receptors, DRD4 appears involved in EDs in a complex manner, being expressed in neurons of the prefrontal cortex and basal ganglia, including the striatum and *nucleus accumbens*, the limbic system and the thalamus [22–24]. DRD4 maps on chromosome 11p15.5 and encodes for a G protein-coupled receptor (GPCR) that inhibits activity of the enzyme adenylyl-cyclase down-regulating neuronal activity. A reduced expression of DRD4 is strongly associated with the risk for Anorexia Nervosa (AN) [25]. Interestingly, a 48 bp VNTR polymorphism (rs1805186) in exon 3 of this gene produces various changes in the length of the receptor protein: among known alleles, the 4-repeat-containing (4R) one produces more efficient receptor proteins and neuronal inhibition than other alleles, such as the 2-repeat (2R) and the 7-repeat (7R) ones, with behavioral consequences that include AN [26, 27] and binge eating disorder (BED) [28].

Together with the enzyme catechol-O-methyltransferase (COMT), the presynaptic transmembrane DA transporter (DAT) represents the neuronal factor responsible for DA deactivation after its synaptic release. DAT is expressed predominantly by neurons located in subcortical structures, including the limbic system and basal ganglia [29], and is, therefore, involved in the regulation of motivated behavior, including feeding. DAT is encoded by the DAT1 gene (SLC6A3) mapped on chromosome 5p15.3. DAT1 has been associated with bulimia nervosa (BN) [30], alcohol withdrawal and dependence [31, 32], and also shown to modify the risk for AN and modulate associated psychopathological features [27]. A 40 bp VNTR polymorphism (rs28363170) is present in the 3′-untranslated region (3′UTR), with most common alleles containing 9- (9R) or 10-repeats (10R) [33]. Although a clear effect of these alleles has not been assessed [34, 35], they appear to produce reduced [36] and increased DAT expression [37, 38], respectively.

Since DA availability in subcortical synapses depending on DAT1 activity may influence DRD4 responses, similarly to other studies carried out on inhibition/impulsivity [39] or in hyperactivity [40], we investigated the possible interaction between the two gene polymorphisms for UE and OE behaviors.

Genetic aspects were studied in association with behavioral features, which were investigated by the Italian adaptation of the Scale for the Assessment of Feeding Interaction (Scala di Valutazione delle Interazioni Alimentari, SVIA) [41] and the Child Behavior Checklist (CBCL) questionnaire [42]. While the SVIA assesses symptoms of feeding-related dysregulation, the CBCL analyzes the presence of externalizing, internalizing and dysregulation symptoms in affective, behavioral and cognitive areas.

## Materials and methods

### Sample

This study, consistent with the Declaration of Helsinki and authorized by the Sapienza Ethical Committee (n. 809/2020), involved 224 3-year-old children and their mothers recruited in Clinical Nutrition Services of pediatric Italian hospitals [*N* = 68 undereating and *N* = 76 overeating] and kindergartens [*N* = 80 normal controls]. Children were matched for age, gender and socio-demographic characteristics of their family. Undereating and Overeating diagnoses were made by pediatricians, based on DC: 0–5 classification criteria [43]. Inclusion criteria for children and mothers were no referred comorbidity with physical or mental disorders, no previous medical or psychological treatment. Eighty-seven duos were excluded due to children’s medical conditions (*N* = 10) and/or comorbidity (*N* = 10 with sensory processing disorder; *N* = 8 with sleep disorder; *N* = 14 with mood disorder; *N* = 9 with obsessive compulsive disorder), or mothers’ medical conditions (*N* = 24), as well as psychiatric diagnoses (*N* = 2 with bipolar disorder; *N* = 10 with anxiety disorder). The final overall sample was composed of 137 children-mother duos, divided according to children’s diagnoses in *N* = 48 UE; *N* = 45 OE; *N* = 44 controls. Mothers were all Caucasian and the biological parent of the children.

All mothers signed the written informed consent and received information about the aims, measures and procedures of the study in preliminary meetings.

### Psychological/behavioral assessments

Mothers helped caregivers completing the Child Behavior Check List (CBCL) 1.5–5 [44], a questionnaire assessing children’s abilities and specific behavior/emotions according to eight subscales (anxious/depressed, withdrawn/depressed, somatic complaints, social problems, thought problems, attention problems, rule-breaking behavior, and aggressive behavior) and two global scales: internalizing problems (with anxious/depressed, withdrawn/depressed, and somatic complaints subscales) and externalizing problems (with rule-breaking behavior and aggressive behavior subscales). The dysregulated profile (DP) (aggregating anxious/depressed, aggressive behavior and attention problems subscales) was also assessed [42].

Specifically trained psychologists evaluated the quality of mother–child interaction during feeding by the Italian adaptation [41] of the Scale for the Assessment of Feeding Interaction [12] (SVIA). The SVIA can be applied to children 1-to-36 months of age and identifies normal and/or risky parent–child interactions after recording for at least 20 min during feeding, coding and evaluated according to 41 items. These are distributed among four subscales, measured on a Likert scale ranging from 0 (none) to 4 (a lot): (a) parent’s affective state (parents’ happiness or sadness/distress during feeding, higher scores referring to greater difficulties in showing positive affection and to more frequent signs of negative affection); (b) interactive conflict (conflictual, non-collaborative and non-empathetic interaction, higher scores referring to parents following their own emotions and intentions rather than children’s signals); (c) food refusal behavior (children’s refusal to open their mouths, being easily distracted and showing opposition or negativity); and (d) Dyad’s affective state (i.e., parents’ and children’s contribution to joy or sadness during feeding, higher scores referring to growing difficulties of caregivers in supporting children’s autonomous initiatives and/or distress and opposition). The inter-evaluator agreement for SVIA items for this sample is excellent (Pearson *r* = 0.9). Moreover, the instrument has good reliability in terms of internal consistency, Cronbach’s *α* = 0.94. The means of the four subscales were also used to derive a total one-dimensional score according to Fadda and Lucarelli (2017) [45], equal to the sum of the four scores [12]. Lucarelli et al. (2002) [41] indicated a cutoff of 54 (2 SDs from the M) to discriminate clinical scores.

### Biological sampling for DNA isolation

Participants’ buccal cell samples were collected by trained personnel through buccal swabs and chilled on ice at 4 °C. DNA was extracted using the Buccal-Prep Plus DNA isolation kit (Isohelix, Cell Projects Ltd., U.K.) according to the manufacturer’s instructions.

### Genotyping

DRD4 rs1805186 genotypes were obtained by PCR amplification using primers with the following sequences: 5′-GCGACTACGTGGTCTACTCG-3′ (DRD4 Forward), 5′-AGGACCCTCATGGCCTTG-3′ (DRD4 Reverse). Reactions were carried out in a final volume of 50 μl with 50 ng DNA, 1.5 mM MgCl_2_, 200 μM dNTPs, 50 mM KCl, 10 mM Tris–HCl (pH 8.3), 0.25 μM primers, 5% DMSO and 1 U Taq DNA polymerase (Promega Corporation Madison WI, USA). An initial denaturation at 95 °C for 3 min was followed by 30 cycles at 94 °C for 45 s, 54 °C for 40 s and 72 °C for 40 s.

DAT1 rs28363170 genotypes were also obtained by PCR amplification using primers with the following sequences: 5′-TGTGGTGTAGGGAACGGCCTGAG-3′ (DAT1 Forward), 5′-CTTCCTGGAGGTCACGGCTCAAGG-3′ (DAT1-Reverse). Reactions were carried out in a final volume of 20 μl with 50 ng genomic DNA, 1.5 mM MgCl2, 200 μM dNTPs, 50 mM KCl, 10 mM Tris–HCl (pH 8.3), 0.25 μM primers and 1 U Taq DNA polymerase. An initial denaturation at 95 °C for 3 min was followed by 30 cycles at 94 °C for 45 s, 57 °C for 30 s and 72 °C for 30 s.

For both polymorphisms, samples were resolved by 3% agarose gel electrophoresis after staining with Syber Safe (Invitrogen Corporation, Waltham MA, USA) and matching to the 100 bp Ladder DNA molecular weight standard (Promega Corporation Madison WI, USA). Genotyping procedures were carried out at least twice for each DNA sample.

Where not stated otherwise, chemicals were purchased from Sigma-Aldrich Co. (St. Louis MO, USA).

### Statistical analysis

Genotype and allele frequencies of DRD4 and DAT1 polymorphisms in EU/OE and control subjects were compared by *χ*^2^ test.

The association of the 2R, 4R and 7R DRD4 genotypes and 9R and 10R DAT1 genotypes with UE and OE and their interaction were further investigated by *χ*^2^ test comparison with the control sample after grouping into three categories: +/+  (homozygous carriers), +/− (heterozygous carriers) or −/− (non-carriers).

The effect of specific genotypes was evaluated by calculation of the odds ratios (OR) with the 95% confidence interval (CI) using the Wilson method [46].

Finally, the scores of the three groups or whole sample on all measures were compared by analyses of variance (ANOVAs). All variables conformed to the assumptions underlying such analyses, including normality of distributions.

A power analysis was conducted according to Cohen’s (2013) [47] suggestions; *α* was set at 0.05 and a power of 0.861 was obtained with a large effect size of (*f*^2^ = 0.45). Sample sizes were calculated according to the cross sectional studies formula of Pourhoseingholi et al. (2013) [48].

All data were analyzed using IBM SPSS statistics, Version 25 (IBM, Armonk, NY, USA).

## Results

### Study sample

Control, UE and OE groups were balanced, overall features being: children’s gender: 69 males, 68 females; children’s and mothers’ ages: 34.8 ± 1.21 months and 33.6 ± 3.4 years, respectively; household income: ≥ 2500 euros, *N* = 120, ≥ 2000 and < 2500 euros, *N* = 17; maternal education, ≥ 13 school years *N* = 123, 12 school years, *N* = 14. Table 1 shows the sample characteristics stratified for sub-groups (Control, UE, and OE).

**Table 1:** Socio-demographic characteristics of the sample sub-groups

	Global	Control	UE	OE	*p*
Children’s gender^a^ (%)	69 males (50.4) 68 females (49.6)	17 males (38.6) 27 females (61.4)	26 males (54.2) 22 females (45.8)	26 males (57.8) 19 females (42.2)	0.16
Children’s age^b^ (months)	34.8 ± 1.21	35.3 ± 2.01	33.2 ± 1.53	35.1 ± 1.36	0.12
Mothers’ age^b^ (years)	33.6 ± 3.4	34.4 ± 2.1	32.1 ± 2.8	34.3 ± 2.4	0.14
Household income	Approximately 2500 euros/month				
Mothers’ education	At least 12 years of schooling				

Values represent means ± standard deviations

^a^Compared by *χ*^2^ test

^b^Compared by Mann–Whitney test

### DRD4 and DAT1 allele frequencies in control, UE and OE groups

DRD4 and DAT1 allele frequencies in the three groups are reported in Table 2.

**Table 2:** Allele frequencies of DRD4 and DAT polymorphisms

Allele	*n* (%)		
	Control	UE	OE
DRD4			
2	8 (9.1)	25 (26.0)^a^	17 (18.9)
3	4 (4.6)	9 (9.4)	8 (8.9)
4	72 (81.8)	29 (30.2)^b^	56 (62.2)^c^
5	3 (3.4)	4 (4.2)	1 (1.1)
6	0	0	1 (1.1)
7	1 (1.1)	27 (28.1)^d^	5 (5.6)
8	0	2 (2.1)	2 (2.2)
DAT1			
3	3 (3.4)	2 (2.1)	5 (5.6)
6	0	0	4 (4.4)
7	8 (9.1)	5 (5.3)	2 (2.2)
9	70 (79.5)	61 (63.5)^e^	25 (27.8)^f^
10	7 (8.0)	26 (27.1)^g^	51 (56.7)^h^
11	0	1 (1.0)	2 (2.2)
12	0	1 (1.0)	1 (1.1)

Only significant differences (*p* < 0.05) are shown:

^a^UE vs. controls: *χ*^2^ (1) = 8.9631, *p* = 0.002755; OR (95% CI) 3.52 [1.49, 8.30] *Z* = 2.88, *p* = 0.004

^b^UE vs. controls: *χ*^2^ (1) = 49.3898, *p* < 0.00001; OR (95% CI) 0.10 [0.05,0.19] *Z* = 6.60, *p* < 0.0001

^c^OE vs. controls: *χ*^2^ (1) = 8.4586, *p* = 0.003633; OR (95% CI) 0.37 [0.18, 0.73] *Z* = 2.86, *p* = 0.004

^d^UE vs. controls: *χ*^2^ (1) = 25.921, *p* < 0.00001; OR (95% CI) 34.04 [4.51, 256.83] *Z* = 3.42, *p* = 0.0006

^e^UE vs. controls: *χ*^2^ (1) = 5.7342, *p* = 0.016638; OR (95% CI) 0.45 [0.23, 0.87] *Z* = 2.37, *p* = 0.018

^f^OE vs. controls: *χ*^2^ (1) = 47.9138, *p* < 0.00001; OR (95% CI) 0.10 [0.05, 0.20] *Z* = 6.54, *p* < 0.0001

^g^UE vs. controls: *χ*^2^ (1) = 11.7342, *p* = 0.000729; OR (95% CI) 4.30 [1.76, 10.51] *Z* = 3.20, *p* = 0.001

^h^OE vs. controls: *χ*^2^ (1) = 48.0629, *p* < 0.00001; OR (95% CI) 15.13 [6.29, 36.39] *Z* = 6.07, *p* < 0.0001

In the control group, 4R was the most frequent allele for the DRD4 polymorphism (81.8%). In the UE group, 2R, 4R and 7R alleles had similar frequencies of 26.0%, 30.2% and 28.1%, respectively. In the OE group, 4R and 2R alleles displayed frequencies of 62.2% and 18.9%, respectively. Differences in allele frequencies between UE and control subjects were observed for the 2R, 4R and 7R alleles (*p* < 0.01). OR evaluation suggests that presence of 2R and 7R alleles represents a risk factor for UE (*p* = 0.004 and *p* = 0.0006, respectively), while that of the 4R allele is a protective factor (*p* = 0.0001). A difference in allele frequency between OE and control subjects was observed only for the 4R allele (*p* < 0.01), also displaying a protective effect (*p* = 0.004). In the control group, 9R was the most represented allele for the DAT1 polymorphism (79.5%). In the UE group, 9R and 10R alleles displayed the highest frequencies (63.5% and 27.1%, respectively). In the OE group, the 9R allele was present in 27.8% of the subjects, while the 10R allele displayed the highest frequency (56.7%). Differences in allele frequencies between UE and control subjects were observed for both 9R and 10R alleles (*p* < 0.05 and *p* < 0.0001, respectively). OR evaluation suggests that while presence of the 9R allele represents a protective factor for UE (*p* = 0.018), that of the 10R allele is a risk factor (*p* = 0.001). A difference in allele frequency between OE and control subjects was also observed for 9R and 10R alleles (*p* < 0.00001), suggesting that the former represents a protective factor (*p* = 0.0001), the latter a risk factor (*p* = 0.0001).

### Genotype frequencies of DRD4 alleles and DAT1 alleles in control, UE and OE groups

We investigated genotype distributions of most frequent alleles, namely 2R, 4R and 7R for DRD4, 9R and 10R for DAT1 in the three groups (Table 3).

**Table 3:** Genotype frequencies of 2R, 4R, 7R DRD4 alleles and 9R, 10R DAT1 alleles

	DRD4			*χ*^2^ (*df* = 2)	*p*
	2R +/+	2R +/−	2R -/−		
Control	3 (6.8)	2 (4.5)	39 (88.6)		
UE	6 (12.5)	13 (27.1)	29 (60.4)	10.383	0.005564
OE	5 (11.1)	7 (15.6)	33 (73.3)	3.767	0.152056
	4R +/+	4R +/−	4R −/−		
Control	35 (79.5)	2 (4.5)	7 (15.9)		
UE	6 (12.5)	17 (35.4)	25 (52.1)	42.386	< 0.00001
OE	20 (44.4)	16 (35.6)	9 (20.0)	15.220	0.00495
	7R +/+	7R +/−	7R −/−		
Control	0	1 (2.3)	43 (97.7)		
UE	2 (4.2)	23 (47.9)	23 (47.9)	28.196	< 0.00001
OE	1 (2.2)	3 (6.7)	41 (91.1)	2.037	0.361201

	DAT1			*χ*^2^ (*df* = 2)	*p*
	9R +/+	9R +/−	9R −/−		
Control	32 (72.7)	6 (13.6)	6 (13.6)		
UE	26 (54.2)	9 (18.8)	13 (27.1)	3.633	0.016263
OE	10 (22.2)	5 (11.1)	30 (66.7)	27.607	< 0.00001
	10R +/+	10R +/−	10R −/−		
Control	1 (2.3)	5 (11.4)	38 (86.3)		
UE	9 (18.7)	8 (16.7)	31 (64.6)	7.643	0.021895
OE	21 (46.7)	9 (20.0)	15 (33.3)	29.298	< 0.00001

Number of subjects: control = 44; UE = 48; OE = 45

All analyses are shown

For all alleles, +/+ indicates homozygous carriers, +/− heterozygous carriers, −/− non-carriers

For DRD4: (i) genotypes containing the 2R allele and 7R allele were more frequent in UE than control subjects (*p* < 0.01 and *p* < 0.00001, respectively); and (ii) genotypes containing the 4R allele were less frequent in UE/OE than control subjects (UE, *p* < 0.00001; OE, *p* < 0.01).

Regarding DAT1: (i) genotypes containing the 9R allele were less frequent in UE/OE than control subjects (UE, *p* < 0.05; OE, *p* < 0.00001); and (ii) genotypes containing the 10R allele were more frequent in UE/OE than control subjects (UE, *p* < 0.05; OE, *p* < 0.00001).

### Dominant and recessive genotype effects of 4R DRD4, 9R and 10R DAT1 alleles in control, UE and OE groups

To dissect the effect of each allele, we analyzed genotype distributions according to dominant or recessive models in the three groups (Table 4).

**Table 4:** Distributions of genotype frequencies of 4R DRD4, 9R and 10R DAT1 alleles according to dominant and recessive models

	DRD4		*χ*^2^ (*df* = 1)	*p*	OR (95% CI)	z	*p*
	4R dominant model						
	+/+ , +/−	−/−					
Control	37 (84.1)	7 (15.9)					
UE	23 (47.9)	25 (52.1)	13.243	0.000274	0.1741 (0.065–0.467)	3.473	0.0005
	4R recessive model						
	+/+	** +/− ,−/−**					
Control	35 (79.5)	9 (20.5)					
UE	6 (12.5)	42 (87.5)	41.770	< 0.00001	0.0367 (0.012–0.113)	5.750	< 0.00001
OE	20 (44.4)	25 (55.6)	11.611	0.000656	0.2057 (0.080–0.526)	3.299	0.0010

	DAT1		*χ*^2^ (*df* = 1)	*p*	OR (95% CI)	z	*p*
	9R dominant model						
	+/+ , +/−	−/−					
Control	38 (86.4)	6 (13.6)					
OE	15 (33.3)	30 (66.7)	25.973	< 0.00001	0.0789 (0.027–0.228)	4.691	< 0.0001
	9R recessive model						
	+/+	+/− ,−/−					
Control	32 (72.7)	12 (27.3)					
OE	10 (22.2)	35 (77.8)	22.771	< 0.00001	0.1071 (0.041–0.282)	4.530	< 0.0001
	10R dominant model						
	** +/+ , +/− **	**−/−**					
Control	6 (13.6)	38 (86.4)					
UE	17 (35.4)	31 (64.6)	5.808	0.0159	3.4731 (1.222–9.872)	2.336	0.0195
OE	30 (66.7)	15 (33.3)	25.973	< 0.00001	12.6667 (4.384–36.594)	4.691	< 0.0001
	10R recessive model						
	+/+	** +/− ,−/−**					
Control	1 (2.3)	43 (97.7)					
UE	9 (18.8)	39 (81.2)	41.770	< 0.00001	9.9231 (1.202–81.929)	2.131	0.0331
OE	21 (46.7)	24 (53.3)	11.611	0.000656	37.6250 (4.760–297.383)	3.439	0.0006

Only significant associations are shown

While no additional information was found for 2R and 7R DRD4 alleles, the 4R DRD4 allele was associated with UE according to both the dominant (*p* < 0.0005) and the recessive model (*p* < 0.00001), suggesting a role as a protective factor (*p* = 0.0005 and *p* < 0.00001, respectively). The 4R allele was also associated with OE according to the recessive model (*p* < 0.001) as a putative protective factor (*p* = 0.001). The 9R DAT1 allele was associated with OE according to the dominant and the recessive model (*p* < 0.00001 for both) as a putative protective factor (*p* < 0.001 for both). The 10R DAT1 allele was associated with UE and OE according to both genetic models (see Table 3 for significance levels) as a putative risk factor.

### Interaction of DRD4 and DAT1 genotypes in control, UE and OE groups

We finally investigated possible gene × gene interactions between DRD4 2R, 4R, 7R alleles and DAT1 9R, 10R alleles. Since receptor activity may depend on synaptic DA availability and, therefore, on DAT1 activity, we evaluated the distributions of DRD4 genotypes in the three groups, sorted according to the dominant and recessive models, in the presence or absence of DAT1 genotypes in the dominant/recessive models as well (Table 5).

**Table 5:** Distributions of genotype frequencies of dominant/recessive model 4R DRD4 allele in the presence or absence of dominant/recessive model DAT1 9R and 10R alleles

	DRD4 4R recessive model				*χ*^2^ (*df* = 3)	*p*
	+/+	+/+	+/− ,−/−	+/− ,−/−		
	DAT1 9R d +	DAT1 9R d −	DAT1 9R d +	DAT1 9R d −		
Control	29 (65.9)	6 (13.6)	9 (20.5)	0		
OE	6 (13.3)	14 (31.1)	9 (20.0)	16 (35.6)	34.307	< 0.00001
	+/+	+/+	+/− ,−/−	+/− ,−/−		
	DAT1 9R r +	DAT1 9R r −	DAT1 9R r +	DAT1 9R r −		
Control	25 (56.8)	10 (22.7)	7 (15.9)	2 (4.6)		
OE	5 (11.1)	15 (33.3)	5 (11.1)	20 (44.4)	29.386	< 0.00001

	DRD4 4R dominant model				*χ*^2^ (*df* = 3)	*p*
	+/+ , +/−	+/+ , +/−	−/−	−/−		
	DAT1 10R r +	DAT1 10R r −	DAT1 10R r +	DAT1 10R r −		
Control	1 (2.3)	36 (81.8)	6 (13.6)	1 (2.3)		
UE	4 (8.3)	19 (39.6)	5 (10.4)	20 (41.7)	24.208	0.00002

	DRD4 4R recessive model				*χ*^2^ (*df* = 3)	*p*
	+/+	+/+	+/− ,−/−	+/− ,−/−		
	DAT1 10R d +	DAT1 10R d −	DAT1 10R d +	DAT1 10R d −		
Control	4 (9.1)	31 (70.4)	2 (4.6)	7 (15.9)		
OE	12 (26.7)	8 (17.8)	18 (40.0)	7 (15.5)	30.357	< 0.00001
	DAT1 10R r +	DAT1 10R r -	DAT1 10R r +	DAT1 10R r -		
Control	1 (2.3)	34 (77.3)	0	9 (20.4)		
OE	10 (22.2)	10 (22.2)	11 (24.4)	14 (31.1)	32.534	< 0.00001

d +, presence of 9R or 10R alleles according to the dominant model (+/+ and +/− genotypes); r +, presence of 9R or 10R alleles according to the recessive model (+/+ genotype); d −, r −, absence of 9R and 10R alleles in either model. Only significant associations are shown

No interaction was observed for 2R and 7R DRD4 alleles (not shown). On the contrary, presence of the DAT1 9R allele, in both dominant and recessive models, displayed interactions with recessive model-DRD4 4R allele on OE (*p* < 0.00001 for both). In the presence of the DRD4 4R allele, non-carriers (d −) and heterozygous carriers (r −) of the DAT1 9R allele were more frequent in OE subjects.

Presence of the 10R allele also displayed an interaction with dominant model-DRD4 4R allele on UE (*p* = 0.00002). Heterozygous carriers (r −) of the DAT1 10R allele were more frequent in control subjects carrying one or two copies of the DRD4 4R allele and more frequent in non-carrier UE subjects. Finally, presence of the DAT1 10R allele displayed interactions with recessive model-DRD4 4R allele on OE (*p* < 0.00001 for both dominant and recessive DAT1 10R models), being more frequent in OE subjects than controls.

### Quality of mother–child interaction in control, UE and OE groups

Mother–child interactions during feeding were inspected by the SVIA and the CBCL scale (Table 6).

**Table 6:** SVIA and CBCL internalizing, externalizing and dysregulated profile (DP) scores

	Control	UE	OE	*F*_(2,134)_
SVIA	26.5 ± 0.2	75.6 ± 1.2	56.3 ± 1.1	48.2^a^
Internalizing	10.3 ± 0.8	29.2 ± 0.3	22.5 ± 1.1	23.5^a^
Externalizing	11.4 ± 0.9	27.3 ± 1.1	25.5 ± 0.6	22.1^a^
DP	13.6 ± 1.3	30.2 ± 1.4	29.1 ± 0.9	32.4^a^

Values represent means ± standard errors

^a^*p* < 0.001, UE or OE vs. Controls, one-way ANOVA

Differences between groups were assessed by one-way ANOVA. In contrast to control dyads, UE and OE dyads exceeded the clinical SVIA cutoff, thus being at risk for the quality of their interactions during feeding. Moreover, UE dyads presented more maladaptive SVIA scores than the other groups. As for CBCL internalizing, externalizing and dysregulation problems, UE and OE children showed significantly higher scores than control children, with UE children displaying the highest scores.

### Quality of mother–child interaction relative to DRD4 and DAT1 genotypes in the whole sample

Specific DRD4 and DAT1 genotype effects on SVIA and CBCL scores, regardless of their involvement in UE and OE, were evaluated in the whole sample by one-way ANOVA (Table 7).

**Table 7:** SVIA and CBCL internalizing, externalizing and dysregulated profile (DP) scores in all children evaluated (*n* = 137), carrying the indicated genotypes regardless of their diagnosis

	SVIA	Internalizing	Externalizing	DP
DRD4 2R				
+/+	57.03 ± 4.96	20.77 ± 1.57	23.14 ± 1.71	23.55 ± 1.49
+/−	64.28 ± 3.48	24.61 ± 1.27	25.06 ± 1.07	25.31 ± 0.90
−/−	50.45 ± 2.09^a^	19.58 ± 0.81^b^	20.31 ± 0.73^c^	21.21 ± 0.62^d^
DRD4 4R				
+/+	40.48 ± 2.25	15.56 ± 0.84	17.36 ± 0.95	18.71 ± 0.8
+/−	64.16 ± 2.37^e^	25.27 ± 0.93^e^	25.11 ± 0.69^e^	25.26 ± 0.6^e^
−/−	62.90 ± 3.0^f^	23.69 ± 1.10^f^	23.99 ± 0.92^f^	24.37 ± 0.79^f^
DRD4 7R				
+/+	71.53 ± 7.87	27.7 ± 2.96	26.5 ± 0.53	26.50 ± 0.53
+/−	74.69 ± 2.32	29.67 ± 0.9^g^	25.92 ± 0.72	26.01 ± 0.65
−/−	47.57 ± 1.8^h^	18.06 ± 0.64^h^	20.08 ± 0.7^i^	21.01 ± 0.6^i^
DAT1 9R				
+/+	49.2 ± 2.79	18.64 ± 1.05	19.31 ± 0.95	20.43 ± 0.81
+/−	56.54 ± 5.12	22.02 ± 1.96	21.79 ± 1.64	22.48 ± 1.4
−/−	57.5 ± 2.09	22.38 ± 0.78^j^	23.94 ± 0.72^k^	24.20 ± 0.62^l^
DAT1 10R				
+/+	60.87 ± 2.02	23.82 ± 0.75	25.23 ± 0.57	25.30 ± 0.51
+/−	59.58 ± 4.01	23.11 ± 1.48	23.84 ± 1.29	24.22 ± 1.09
−/−	49.02 ± 2.45^m^	18.65 ± 0.92^n^	19.33 ± 0.84^o,p^	20.42 ± 0.71^o,q^

Values represent means ± standard errors

One-way ANOVA: DRD4 2R—SVIA: *F*_2,137_ = 4.38; *p* = 0.014; internalizing: *F*_2,137_ = 3.77; *p* = 0.026; externalizing: *F*_2,137_ = 4.54; *p* = 0.012; DP: *F*_2,137_ = 4.63; *p* = 0.011

DRD4 2R, multiple comparisons vs.  +/−: ^a^*p* = 0.019; ^b^*p* = 0.026; ^c^*p* = 0.020; ^d^*p* = 0.018

One-way ANOVA: DRD4 4R—SVIA: *F*_2,137_ = 30.02; *p* = 0.000; internalizing: *F*_2,137_ = 32.23; *p* = 0.000; externalizing: *F*_2,137_ = 22.35; *p* = 0.000; DP: *F*_2,137_ = 22.34; *p* = 0.000

DRD4 4R, multiple comparisons vs. +/+: ^e^*p* = 0.000; vs. +/+: ^f^*p* = 0.000

One-way ANOVA: DRD4 7R—SVIA: *F*_2,137_ = 26.55; *p* = 0.000; internalizing: *F*_2,137_ = 37.50; *p* = 0.000; Externalizing: *F*_2,137_ = 8.62; *p* = 0.000; DP: *F*_2,137_ = 8.76; *p* = 0.000

DRD4 7R, multiple comparisons vs. +/+: ^g^p = 0.036; vs.  +/−: ^h^*p* = 0.000; ^i^*p* = 0.001

One-way ANOVA: DAT1 9R—SVIA: internalizing: *F*_2,137_ = 3.87; *p* = 0.023; externalizing: *F*_2,137_ = 6.47; *p* = 0.002; DP: *F*_2,137_ = 5.92; *p* = 0.003

DAT1 9R, multiple comparisons vs. +/+: ^j^*p* = 0.037; ^k^*p* = 0.002; ^l^*p* = 0.004

One-way ANOVA: DAT1 10R—SVIA: *F*_2,137_ = 5.16; *p* = 0.007; internalizing: *F*_2,137_ = 6.85; *p* = 0.001; externalizing: *F*_2,137_ = 10.41; *p* = 0.000; DP: *F*_2,137_ = 9.91; *p* = 0.000

DAT1 10R: ^v^multiple comparisons vs. +/+: ^m^*p* = 0.021; ^n^*p* = 0.006; ^o^*p* = 0.000; vs.  +/−: ^p^*p* = 0.029; ^q^*p* = 0.031

In line with observations on effects of various alleles on UE and OE groups, subjects carrying the DRD4 2R and 7R alleles displayed values that exceeded the clinical cutoffs of the SVIA and CBCL scores when compared to 4R homozygotes, who instead displayed scores below the clinical significance. As for DAT1 genotypes, 9R homozygotes showed the lowest scores for the SVIA and CBCL scores when compared to 9R heterozygotes and 10R allele-containing genotypes.

## Discussion

The present results suggest that DRD4 and DAT1 genes: (i) have a role in abnormal feeding in young children; (ii) affect the general quality and specific aspects of mother–child interaction during feeding; (iii) influence each other, allowing to confirm a proposed functional mechanism that regulates DA dynamics underlying specific phenotypic effects. As such, they extend observations already reported for these genes on ADHD [40, 49] and impulsivity [39].

### Effects of DRD4 alleles on UE and OE behavior

The DRD4 7R allele was associated with UE behavior as a putative risk factor, in line with the hypothesis that DA dysfunction is among neuronal mechanisms that increase susceptibility to AN in adolescents and adults [26, 27]. The 2R and 4R alleles appeared to affect eating behavior as well, the former being more frequent in UE children, the latter less frequent in UE and OE children.

DRD4 is a postsynaptic GPCR that lowers the amount of endogenous cAMP, producing neuronal repression. Since alleles with 7 or more repeats have reduced expression when compared with shorter ones [25, 50], in the presence of the 7R allele both in homozygous and heterozygous carriers, neurons may be not efficiently inhibited by DA release. This would represent the possible functional effect of this allele, as a risk factor, on the UE phenotype. According to Volkow et al. (2004) [51], a decrease in DA response is central to attentional impairment in ADHD and altered neuronal repression in 7R allele carriers is among the causes of hyperactivity [52, 53]. We favor this functional hypothesis on impaired neuronal inhibition produced by the 7R DRD4 allele and suggest that the resulting increased activity of DA neurons is also among the neurobiological causes of reduced feeding in small children, via an attentional impairment and easy distractibility.

Presence of the 2R allele also appears to behave as a moderate risk factor for UE, in agreement with the observation that the 2R and 7R alleles provide reduced efficiency in signal transduction and neuronal inhibition when compared to the 4R allele [54]. Additional investigation on this allele is warranted to better understand its functional effect.

Conversely, the 4R allele is strongly suggested to represent a protective factor for both UE and OE. Interestingly, this allele appears to represent a case of incomplete dominance, its effects being stronger in homozygotes then in heterozygotes.

### Effects of DAT1 alleles on UE and OE behavior

Our analyses suggest that both 9R and 10R alleles of DAT1 have a role in early EDs, the former as a protective factor, the latter as a risk factor. DAT1 has been associated with attention, since amounts of synaptic DA appear to influence neuronal inhibition necessary to reach a “normal” attentional phenotype [55], regardless of the efficiency of receptors. Due to its reduced expression [36], the DAT1 9R allele, by keeping synaptic DA levels high, may affect attention positively and this may contribute to normal feeding. On the contrary, the DAT1 10R allele, producing increased DA reuptake [37, 38], may further compromise the response of postsynaptic neurons, thus representing a risk factor for EDs. Interestingly, both DAT1 alleles affect OE. Since striatal DA pathways may be involved in altered neural bases of reward process in EDs, DAT1 10R allele carriers, due to reduced amounts of synaptic DA, would be more sensitive to reward reinforcement of food. DAT1 10R carriers with low-quality mother/child feeding interactions (see Table 7) might therefore consume higher amounts of food to regulate DA availability and reward, leading to overeating. Anyhow, the functional mechanism for these effects needs further investigation.

### Interaction of DRD4 and DAT1 alleles on UE and OE behavior

The DRD4 4R allele appeared to interact with DAT1 9R and 10R alleles, supporting previous observations on DA mechanisms in the pathogenesis of psychopathology [56]. Present observations reinforce the role of DRD4 [40, 57, 58] and DAT1 [59] in psychopathology and further support the idea that activity of DRD4 is influenced by DAT-dependent synaptic DA availability, with visible behavioral effects.

### Quality of mother–child interaction and effects of DRD4 and DAT1 alleles

Consistent with previous literature that demonstrates how the low quality of parent–child exchanges during feeding is closely associated with child’s disordered eating [60], SVIA and CBCL scores showed that dyads with undereating and overeating children have maladaptive and clinically at-risk interactions during feeding. Noteworthy, dyads with undereating children display more maladaptive SVIA scores than the other groups, also confirms that undereating in offspring can be associated with severe impairment in the relationship with parents, particularly with the mother [61]. Moreover, UE and OE children showed significantly higher internalizing, externalizing and dysregulation symptoms than control children, UE children having the highest scores. It is recognized that children with eating disturbances have higher risk for psychopathology, or other psychopathological symptoms in comorbidity [61, 62]. Our results extend comorbidity to symptoms of dysregulation and strongly suggest that UE children have more severe relational problems than OE children.

Association of DRD4 2R and 7R alleles with higher scores in both the SVIA and CBCL scales suggests that they also represent a risk factor for these relational aspects. On the contrary, association of the DRD4 4R allele with lower scores in both the SVIA and CBCL scales suggests a protective role for this allele. In agreement with results on undereating or overeating behavior, the DAT1 9R allele appears to be associated with lower SVIA and CBCL scores, suggesting a protective role, while the DAT1 10R allele is associated with higher scores and is a putative risk factor.

In sum, our study presents associations between variants of the DRD4 and DAT1 genes, children’s eating behavior and mother–child interactions. Although no causal conclusions can be drawn, we would hypothesize that DRD4 and DAT1 have a role in children’s neuronal responses necessary for a normal relationship with their mothers during feeding and on their eating behavior.

### Strength and limits

Although not performed on a large sample, this study has two major strengths: (a) all enrolled children had a clinical diagnosis performed by pediatricians, based on the DC:0–5 Classification criteria; (b) mother–child interactions during feeding were objectively investigated by the SVIA and analyzed by clinicians; (c) the consistent direction in the association of the genetic data with different behavioral features, all representing markers of psychopathology, makes the possibility that they are due to spurious correlations highly unlikely.

### What is already known on this subject?

While a growing body of information is now available on the genetics of major EDs in adults, data on young children are still missing. Studies on genetic and psychological variables associated with abnormal behavior in infancy would represent new tools for Developmental Psychologists, considering their interest in children’s EDs.

### What this study adds?

This study provides a first evidence of a role for DRD4 and DAT1 genetic polymorphisms, in addition to specific psychological variables, in abnormal eating behavior in the first years of life, with a potential strong neurobiological and clinical impact in early psychopathology.

## Data Availability

The datasets generated during and/or analyzed during the current study are available from the corresponding author on reasonable request.
